# Draft genome sequencing and secretome profiling of *Sclerotinia sclerotiorum* revealed effector repertoire diversity and allied broad-host range necrotrophy

**DOI:** 10.1038/s41598-022-22028-z

**Published:** 2022-12-17

**Authors:** Navin C. Gupta, Sunita Yadav, Shaweta Arora, Dwijesh C. Mishra, Neeraj Budhlakoti, Kishore Gaikwad, Mahesh Rao, Lakshman Prasad, Pramod K. Rai, Pankaj Sharma

**Affiliations:** 1 ICAR-National Institute for Plant Biotechnology, New Delhi, India; 2 Division of Agricultural Bioinformatics, ICAR-Indian Agricultural Statistics Research Institute, New Delhi, India; 3 ICAR-Indian Agricultural Research Institute, Pusa, New Delhi, India; 4 ICAR-Directorate of Rapeseed-Mustard Research, Bharatpur, Rajasthan India

**Keywords:** Genome informatics, Pathogens, Microbiology, Fungi, Fungal genomics

## Abstract

White mold commonly known as *Sclerotinia sclerotiorum* causes stem rot disease and has emerged as one of the major fungal pathogens of oilseed *Brassica* across the world. In the present study, consistently virulent *S. sclerotiorum* isolate “ESR-01” was sequenced and an assembly size of ~ 41 Mb with 328 scaffolds having N50 of 447,128 was obtained. Additionally, 27,450 single nucleotide polymorphisms (SNPs) were identified from 155 scaffolds against *S. sclerotiorum* 1980 isolate, with an average SNP density of ~ 1.5 per kb genome. 667 repetitive elements were identified and approximately comprised 7% of the total annotated genes. The DDE_1 with 454 in numbers was found to be the most abundant and accounts for 68% of the total predicted repetitive elements. In total, 3844 simple sequence repeats are identified in the 328 scaffolds. A total of 9469 protein-coding genes were predicted from the whole genome assembly with an average gene length of 1587 bp and their distribution as 230.95 genes per Mb in the genome. Out of 9469 predicted protein-coding genes, 529 genes were observed encoding the CAZymes (Carbohydrate-Active enzymes) capable of degradation of the complex polysaccharides. Glycosyltransferase (GT) families were most abundant (49.71%) among the predicted CAZymes and GT2 (23%), GT4 (20%), and glycoside hydrolase (GH) 23% with GH18 (11%) were the prominent cell wall degrading enzyme families in the ESR-01 secretome. Besides this, 156 genes essential for the pathogen-host interactions were also identified. The effector analysis in the whole genome proteomics dataset revealed a total of 57 effector candidates (ECs) and 27 of them were having their analogs whereas the remaining 30 were novel ones. Eleven selected ECs were validated experimentally by analyzing the expression profile of the ESR-01 isolate of *S. sclerotiorum*. Together, the present investigation offers a better understanding of the *S. sclerotiorum* genome, secretome, and its effector repertoire which will help in refining the present knowledge on *S. sclerotiorum*-*Brassica* interactions and necrotrophic lifestyle of the phytopathogen in general.

## Introduction

Constantly evolving broad generalist fungal pathogen *Sclerotinia sclerotiorum* infects a wide array of plant species including oilseed *Brassica* (*B. napus, B. rapa, and B. juncea*), soybean (*Glycine max*), garden lettuce (*Lactuca sativa*), peanut (*Arachis hypogaea*), sugar beet (*Beta vulgaris*), and many other agronomically important crops accounting more than 500 plant species of 278 genera belonging to 75 families^1–5^. The polyphagous nature and ubiquitous presence of this pathogen make it a major biotic factor that causes severe annual losses in terms of yield and seed quality at the pre-and post-harvest stages of oilseed *Brassica* cultivation. *Brassic*a species are the third largest edible oil source in the world after soybean and palm oil. The disease incidence of stem rot on *Brassica* has increased at an alarming rate taking its toll in almost all the *Brassica* growing countries including India, the USA, Canada, and Australia. India is the third-largest producer of *Brassica* oilseed and simultaneously it is also ranking 7th largest importer of edible oil in the world. Sclerotinia stem rot disease is the major biotic stress of the *Brassica* in the Indian context and it mostly prevailed in the north-western part of India, especially in Rajasthan, Haryana, Punjab, Uttar Pradesh Bihar, Madhya Pradesh, and others^6,7^. The destructive nature of this pathogen is more pronounced during the flowering stages where the infection starts carpogenically as ascospores infect either directly or wind-blown to leaves or onto the stem area. Therefore, comprehensive knowledge of pathogen biology and its infection mechanism is the key feature determining the fate of the developing strategies to control the disease spread in the field. The infection behavior of *S. sclerotiorum* makes it be considered a hemibiotroph otherwise earlier it was considered a necrotrophy^8,9^. Necrotrophs are less adapted and secrete effectors that kill the infected region of the host to feed on them for their survival whereas hemibiotrophs and biotrophs, adopt diverse lifecycle during evolution based on their infection strategy to keep the host live and proliferate along with them^10^. They secrete specialized effector protein that suppresses plant defense and manipulates host physiology to obtain nutrition and promotes infection^11^. Several pathological and molecular studies are being carried out to understand the nature and the pathogenic variability in *S. sclerotiorum* isolates’ but these are still at their infancy stage^1^. Host plants are also evolving and getting smarter in terms of host defense against fast-evolving pathogens^12^. The pace of co-evolution is very fast, so to catch it up, large-scale genomic studies are needed to understand the sources of variation. Although *S. sclerotiorum* strain 1980 has already been sequenced^13^ and re-sequenced^14^, it is evidenced by the field evaluation study on the comparative pathogenicity of *S. sclerotiorum* (CCS HAU-Hisar) strain against *Brassica* germplasm (*B. juncea and B. napus*) of Indian, Australian and Chinese origin, revealed that isolates virulence varies with different hosts^15^. In our previous study, the evolutionary relationship between genetic diversity and pathogenicity differences among sixty-five geographically distinct isolates of *S. sclerotiorum* indicates that the aggressiveness of the isolates varies with varying *Brassica* species^1^. Differential aggressiveness of isolates can be attributed to variable genetic, and molecular attributes leading to differential effector repertoire among isolates. The life-cycle of the pathogen and its host specificities are the key determinants for effector divergence. To acquire an in-depth understanding of the pathogenesis and infection mechanisms of necrotrophic fungi, the necrotrophy-associated genomic characteristics and sequences of the effector repertoire need to be analyzed thoroughly. ESR-01, one isolate among 65 *S. sclerotiorum* collections, has been observed to be consistently pathogenic to almost all the tested *Brassica* germplasm. Based on the consistency in virulence, ESR-01 was selected for the whole-genome sequencing and comprehensive analysis with deciphering the genetic sources of variation and its pathogenicity factors.

The virulence of the pathogen usually varies with their isolates of different origins, interacting host and environmental factors. So far, the genomics and transcriptomics studies on *S. sclerotiorum* pathogen reported are based on their interaction with the *B. napus*, however, the present study is on an Indian isolate of the *S. sclerotiorum* ‘ESR-01’ found pathogenic across the *Brassica* germplasm. The recent advances in sequencing techniques and bioinformatics tools make analyzing the data swiftly and more informative in terms of unraveling the virulence-associated genes, effectors, transporters, signaling cascades, regulatory molecules, epigenetic factors those interplay in host–pathogen interaction along with the genetic diversity factors. The variation in SSRs among the isolates of the same species helps in determining genetic and evolutionary relationships across the species. Similarly, SNPs are the functional variant that alters phenotype through alteration of molecular function and thus the association of pathogen with virulence can also be explored. The secretome analysis from the whole genome sequence involves the identification of genes associated with secretory protein and their association with virulence, host–pathogen interaction, and host defense response. Some of the secretory protein bears the polysaccharide degradation mechanism predicted as Carbohydrate-Active enZYmes (CAZymes) which facilitates pathogen entry to the host cell.

This study reports the draft genome sequence of *S. sclerotiorum* “ESR-01”, an Indian isolate, and its secretory effector repertoire. We also reported the prediction of the genome-wide distributed transposable elements, SSRs, and SNPs. Through secretome analysis and annotation, the effector repertoire of *S. sclerotiorum* was predicted to gain insight into the effectors related to necrotrophy. The predicted secretory proteins were also searched for homology with experimentally validated pathogenesis-related genes in PHI-base. The predicted effector proteins, CAZyme, and PHI-base repertoire of *S*. *sclerotiorum* would be useful in identifying their target molecules that will help in designing stringent control measures for this devastating but poorly characterized pathogen in Indian mustard (*Brassica juncea*).

## Results

**Figure 1:**
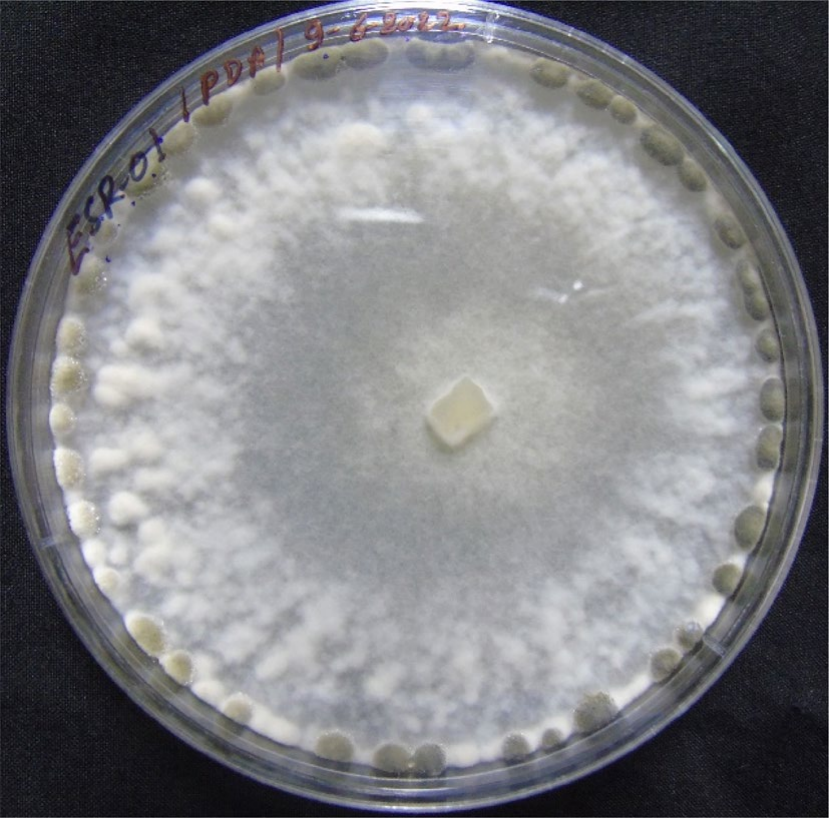
The *S. sclerotiorum* ‘ESR-01’ isolate from the *Brassica juncea* field of Rajasthan, India (Long. 77.300° E; Lat. 27.150° N) was used for whole-genome sequencing.

The secretory proteins or effector molecules of the pathogen and their interaction with the host defense mechanism are the key determinants of virulence and the resistance/susceptible reaction in the host, respectively. These are further influenced by the external environmental factor and controlling the pathogenesis processes. Although genomic content may not differ much in different isolates of the same species, the virulence determining factor varies due to changes in the host physical parameters and the prevailing external conditions. The approach toward revealing the genome-wide secretory proteins for identifying the disease-causing factors can help in understanding the dynamic nature of the pathogen’s genomic features and their virulence strategies. The *S. sclerotiorum* isolate “ESR-01” (Fig. 1) belonging to a virulent category and clad-I of their genetic diversity as described in previous studies^1^ was sequenced and analyzed.

### Genomic features

**Figure 2:**
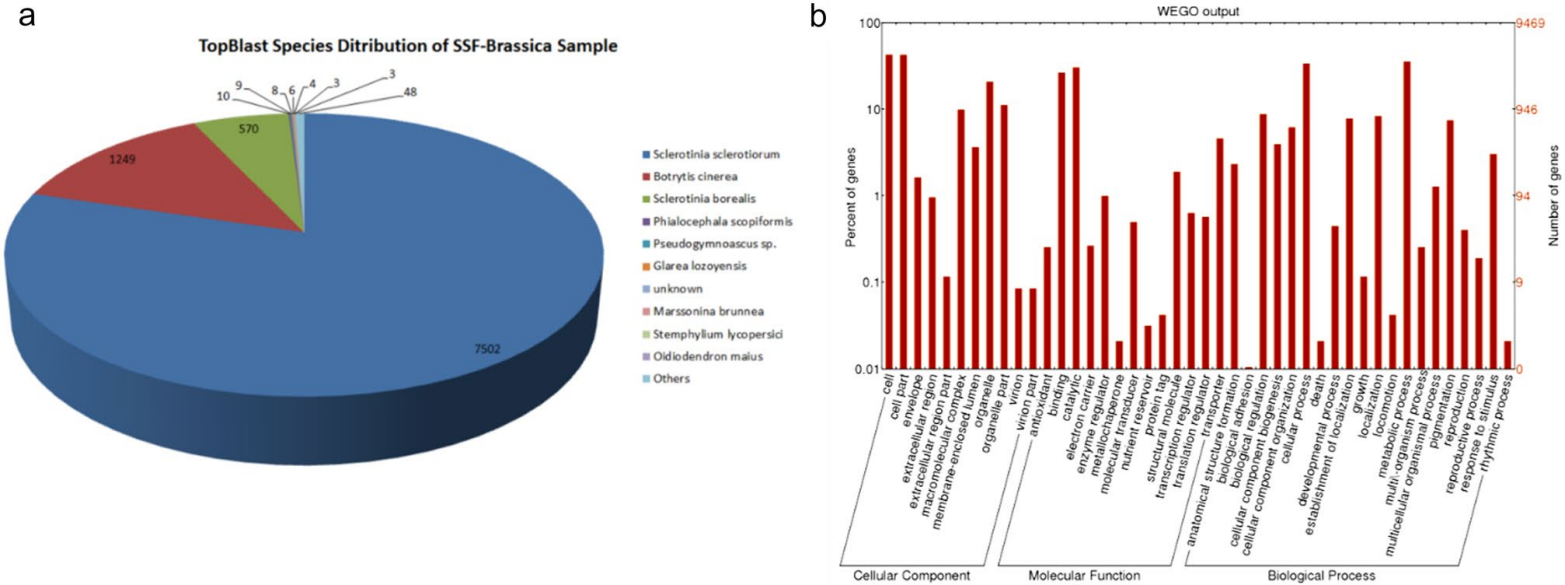
Genome sequence feature of the *S. sclerotiorum* ‘ESR-01’ isolate. (**a**) Top Blast Species distribution chart and (**b**) WEGO plot for the GO term distribution of the genes predicted in whole-genome sequences of ‘ESR-01’ isolate into biological process, cellular component, and molecular function.

The specificity of the ‘ESR-01’ isolate was confirmed by nucleotide homology for the ITS4 region with PCR amplification by using the species-specific primers (Table S1). A paired-end shotgun library with a mean fragment size of 478 bp yielded ~ 6.9 GB, while a mate-pair library with a mean fragment size of 818 bp yielded ~ 3.5 GB of sequencing data with clean reads. The filtered high-quality reads, paired-end (PE) and mate-pair (MP) read of 23,191,545 and 11,569,965, respectively, were de novo assembled into 328 scaffolds (BioProject PRJNA722876; WGS Accession No. JAGTAE010000000), with an N50 scaffold size of ~ 447.13 kb using SOAPdenovo (v2.04) (Table S2). Assembly resulted in a total size of 40.98 Mb with an overall coverage of 129X (Table 1). Around 80% of the scaffolds were larger than 5 kb including 35% of scaffolds larger than 50 kb (Table S3). In total 9469 protein-coding genes were predicted from assembled scaffold against *Botrytis cinerea* using the AUGUSTUS-3.2.1 gene prediction program. The average gene density in the *S. sclerotiorum* genome was 231 genes per Mb and the average gene length of the predicted gene was 1587 bp (Table 1), while the genes between 1 and 5 kb in length were most abundant and accounted for 6019 genes (Table S4). The overall GC content of the *S. sclerotiorum* (ESR-01) genome was estimated to be 37.71% whereas the GC content of the euchromatic region (gene-rich) was 45.88%. Other than the 9469 protein-coding genes, 74 tRNA and 11 rRNA coding genes were also predicted (Table 1). Functional annotation with the BLASTx program (NCBI-blast-2.3.0 + standalone tool) has shown the homologous sequences for 9412 genes against NR (non-redundant protein) database whereas the remaining 57 genes were without any blast hits (Table 1). The majority of the hits were found with the *S. sclerotiorum* genome ~ 81% (7502 genes) followed by ~ 13% (1249 genes) with *B. cinerea* and approximately 6% (570 genes) with *S. borealis* (Fig. 2a). Gene ontology (GO) mapping was carried out by using the Blast2GO PRO and that revealed 4514 genes involved in biological processes, 4677 genes in molecular processes, and 4151 genes were assigned to cellular functions (Fig. 2b). Further, the potential involvement of the 2994 predicted genes in different KEGG pathways was established (Table 2). All the genes were classified mainly under five categories, metabolism, cellular process, genetic information processing, environmental information processing, and organismal system.

**Table 1:** Assembly and gene statistics features of *Sclerotinia sclerotiorum* isolate ‘ESR-01’.

Features	*S. sclerotiorum* isolate “ESR-01”
Coverage	129 X
Assembly size (Mb)	40.98
Scaffold N50	447,128
GC content (%)	37.71
Predicted protein-coding genes	9469
Average gene length (bp)	1587
Gene density (number of genes per Mb)	231
Unique genes	57
GC content in predicted genes (%)	45.88
tRNA genes	74
rRNA gene	11
Transposable elements	647
Secreted proteins	944
SNPs	27,450
SSRs	3844

**Table 2:** KEGG Pathway analysis of the predicted genes of *S. sclerotiorum* ‘ESR-01’ isolate.

Pathway categories	Number of genes
**Metabolism**	
Overview	207
Carbohydrate metabolism	282
Energy	143
Lipid	140
Nucleotide	105
Amino acid	225
Metabolism of other amino acids	73
Glycan biosynthesis and metabolism	67
Metabolism of cofactors and vitamins	124
Metabolism of terpenoids and polyketides	28
Biosynthesis of other secondary metabolites	39
Xenobiotics, biodegradation, and metabolism	66
**Genetic Information processing**	
Transcription	138
Translation	292
Folding, sorting, and degradation	233
Replication and repair	96
**Environmental information processing**	
Membrane transport	7
Signal transduction	231
Signaling molecules and interactions	2
**Cellular process**	
Transport and catabolism	261
Cell motility	23
Cell growth and death	154
Cellular community-eukaryotes	37
**Organismal system**	
Environmental adaptation	21

### Secondary metabolite profile of *S. sclerotiorum* ‘ESR-01’ isolate

The AntiSMASH analyses of the *S. sclerotiorum* ‘ESR-01’ isolate revealed nine NRPS, five type-I PKS, and one terpene gene cluster. Two of the five type-I PKS clusters produces known secondary metabolite, viz*.* botcinic acid, naphthalene, and squalestatin S1 (Table S5). Botcinic acid has a redundant role in virulence along with the botrydial. The cluster present in scaffold 3 of the ESR-01, contains 16 homologs genes of *B. cinerea B05.10* botcinic acid gene cluster (*Bcboa* 1-*Bcboa* 17). In this cluster, there were two core biosynthetic genes (*Bcboa 6* & *Bcboa* 9), 5 additional biosynthetic genes (*Bcboa*-3, 4, 5, 7, and 17), and 9 other genes (Fig. S1a) were present. The second known cluster in scaffold 11 is homologous to nine genes encoding naphthalene from *Daldinia eschscholzii IFB-TL01*. The cluster found has one core biosynthetic gene (gene 6), 1 additional biosynthetic gene (gene 4), and 7 other genes (Fig. S1b). The third cluster present in scaffold 47 encodes a phytoene synthase involved in the synthesis of a fungal metabolite Squalestatins S1, a terpene compound serving as an inhibitor of the squalene synthase. The terpene cluster found has one core biosynthetic gene (gene 2) and four other genes which are homologous to five genes from *Aspergillus sp*. Z5. (Fig. S1c).

### Transposable elements, SSR and SNP

**Figure 3:**
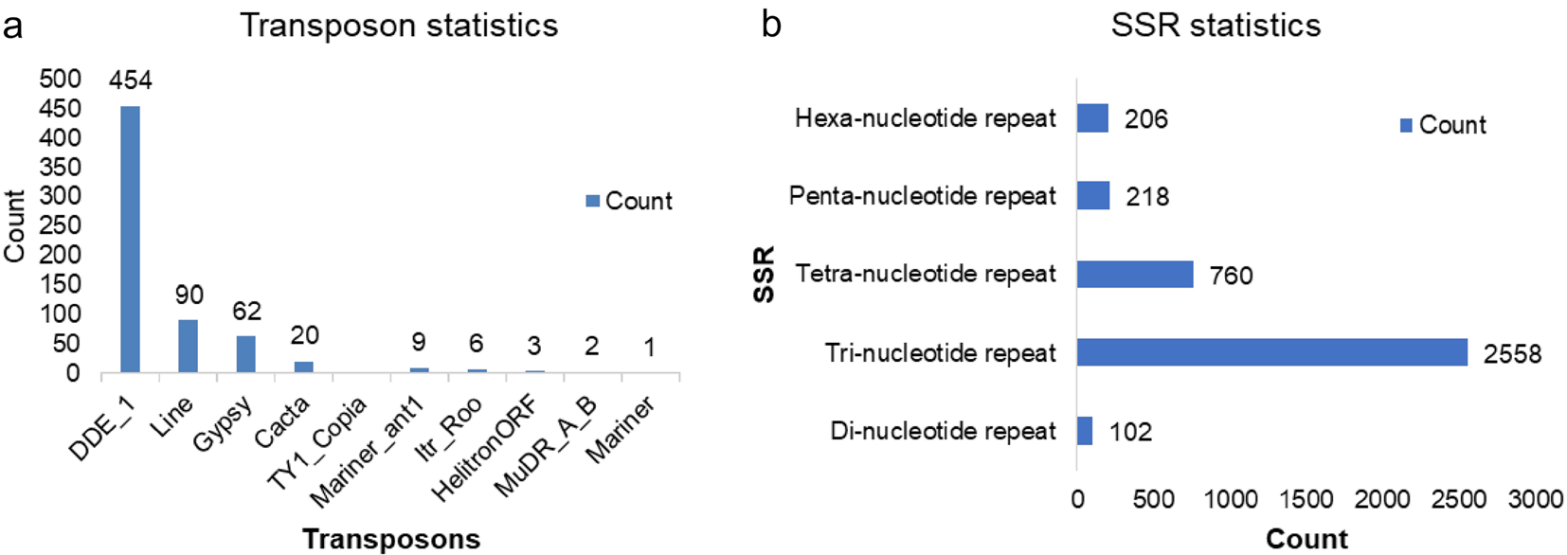
Abundance and distribution of the repeated elements in *S. sclerotiorum ‘*ESR-01’ isolate. (**a**) transposable elements (**b**) SSR markers.

667 repeats/transposable elements were predicted from 155 scaffolds of the genomic assembly. These repeat elements were classified into 10 different families among which DDE_1 was the most abundant having 454 elements followed by the “LINE” family having 90 repeat elements and “gypsy” having 62 elements (Fig. 3a).

SSRs, also known as microsatellites, are tandem repeated motifs of 1–6 bases and serve as the most important co-dominant markers in population and conservation genetics, and gene mapping. Conservation genetics of SSRs was studied as it creates and maintains genetic variation and in turn play important role in genome evolution. Including 318 compound SSRs, 3844 SSRs were predicted from the assembled scaffolds by using the software MISA (Fig. 3b). Tri-nucleotide repeats were the most abundantly present (2558), followed by a tetra-nucleotide repeat with 760 SSRs. Further, a total of 27,450 SNPs were detected in the genome and among them, 26,991 were homozygous SNPs and the others were heterozygous.

### Secretome analysis

**Figure 4:**
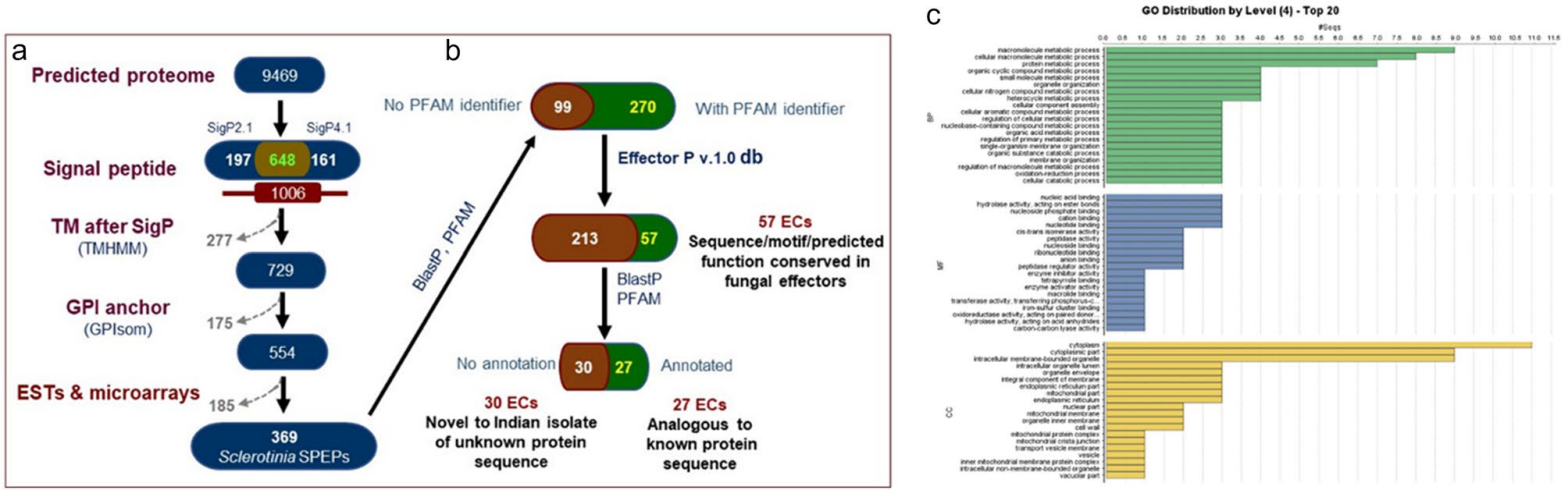
*S. sclerotiorum* secretome prediction, effector identification, and WEGO plot depicting the distribution of the top 27 hits of the predicted effector proteins (**a**) Out of the 554 predicted secreted proteins, 369 had experimental evidence for expression *in planta* (*S. sclerotiorum* secreted proteins expressed *in planta*, SPEPs). The number of proteins filtered out is indicated with dotted arrows, the number of selected proteins is given within boxes, bioinformatics tools and resources used are indicated by the boxes (**b**) identification of effector candidates (ECs) based on sequence, motifs, or protein domains conserved in fungal effectors, and (**c**) The results are summarized in three main GO categories, cellular components (CC), molecular function (MF) and biological processes (BP). The left y-axis indicates the percentage of a specific category of genes in that main category.

Of the 9469 predicted proteins in the ESR-01 genome, a total of 1006 proteins were annotated as classical secretory proteins by SignalP versions 2.1 and 4.1. Out of the 1006 predicted secretory proteins. 648 were commonly classified by these prediction tools, whereas 197 and 161 proteins were exclusively classified by the signal IP2.1 and 4.1, respectively. The 1006 proteins were further scanned with TMHMM and 729 sequences were predicted as secretory proteins after the removal of 277 transmembrane proteins from the protein data set. The filtered 729 proteins were further screened using the GPI-SOM server and 167 proteins with glycosylphosphatidylinositol (GPI) anchor along with 8 peptides as unclassified proteins were removed. The remaining 554 secretory proteins were filtered by pBLASTN against the available EST and microarray sequences of *B. napus* infected with *S. sclerotiorum* resulting in 369 blast hits (Fig. 4a). Further, these 369 predicted effectors were searched against the PFAM database resulting in 99 peptides with no identifier of the PFAM family. The remaining 270 genes were further analyzed using the EffectorP protein server resulting in 57 effectors proteins (Fig. 4b). Annotation of these 57 protein sequences using PFAM showed only 27 proteins with their identity and description (Table S6). The remaining 30 effector proteins were novel (Table S6) and need to be further evaluated for their role in pathogenesis. Secretory effectors (57) were functionally annotated and mapped using the Blast2GO PRO to categorize them into GO terms. The involvement of these predicted 57 effectors in the biochemical pathway was deciphered by KEGG analysis. The results showed only 4 secretory effectors were involved in the distantly related pathway of pathogenesis and those were related to purine metabolism, glyoxylate and dicarboxylate metabolism, drug metabolism, and T cell receptor signaling pathway along with riboflavin and thiamine metabolism (Fig. 4c).

### Polysaccharide degradation machinery and gene families involved in pathogenicity

**Figure 5:**
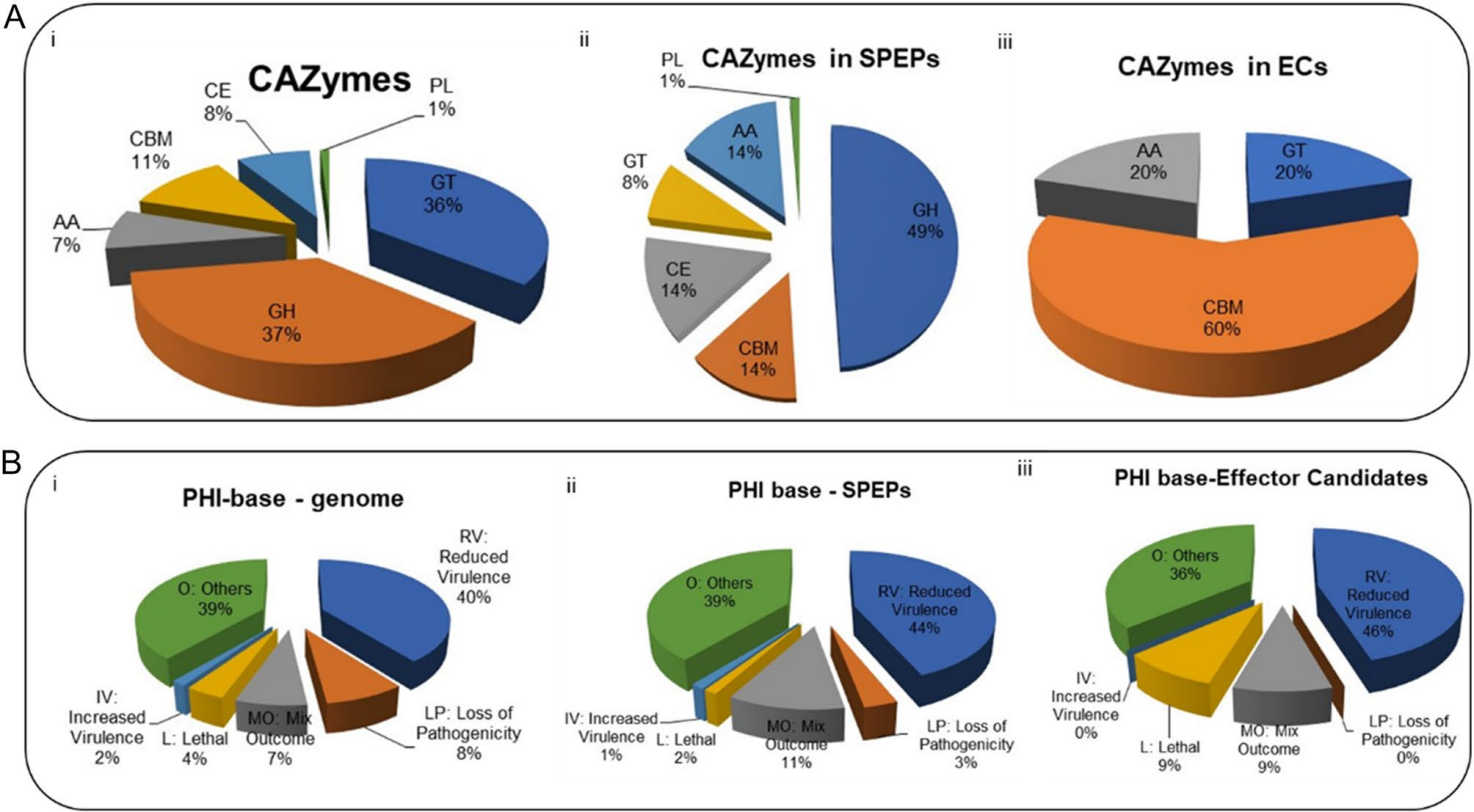
Distribution pattern of the predicted CAZymes (**A**) & pathogen-host interacting factors (**B**) in *S. sclerotiorum ‘*ESR-01’ isolates. (Ai) Summary of the six CAZymes categories: carbohydrate-binding modules (CBMs), Glycosyltransferases (GTs), glycoside hydrolases (GHs) polysaccharide lyases (PL), carbohydrate esterase (CE), and auxiliary activities (AA) (Aii) Distinct summaries of the CAZyme present in SPEP (Secreted proteins expressed *in planta*) (Aiii) Distinct summaries of each of the CAZyme present in Effector Candidates (**B**) Summary of different phenotypic categories of orthologs genes of *S. sclerotiorum ‘*ESR-01’ isolate based on the pathogen-host interactions (PHI-base) indicated in (i) genome (ii) SPEPs, and (iii) Effector Candidates.

CAZyDB tool was used to predict CAzymes involved in polysaccharide degradation or modification. Among the 9469 genes, 1506 were predicted to be CAZyme and functionally classified into 6 categories namely glycosyltransferases (GT), glycoside hydrolases (GH), polysaccharides lyases (PL), carbohydrate esterase (CE), carbohydrate-binding modules (CBM), and auxiliary activities (AA). The CAZymes predicted among the genes further fall under 163 enzyme families. Amongst which GH was most abundant and found to be present in the 550 genes followed by GT class to be present in 542 predicted genes (Fig. 5Ai). Within these classes GT2 was most abundant and present in 121 genes, followed by GT4 (108 genes) and GH18 (60 genes) while the rest of all families spanned less than 50 genes.

The prediction of CAZymes in a total of 369 predicted secretory proteins showed only 164 genes carrying the CAZyme properties and were mostly spanned by glycoside hydrolase where the GH28 family was the abundant one (Fig. 5Aii; Table S7). Further, among the 57 predicted effector candidates, only 5 genes were found to have CAZyme and only three of them had the CBM class of CAZyme in which the CBM13 family was observed abundantly present (Fig. 5Aiii; Table S7).

Homology search in 9469 predicted genes for the potential pathogenicity-related genes showed 2765 genes were aligned to PHI-base proteins at 1e^−05^. These pathogenesis-related proteins were distributed majorly into six categories based on their functional attributes and characteristic features. These categories include hypervirulence, reduced virulence, loss of pathogenicity, lethal, mixed outcomes, and others as shown in Fig. 5Bi. Among 369 secretory proteins, 153 were grouped into these categories using PHI-base (Fig. 5Bii) whereas only 11 out of 57 secretory effectors were aligned in PHI-base (Fig. 5Biii; Table S8).

### Secretome diversity analysis

**Figure 6:**
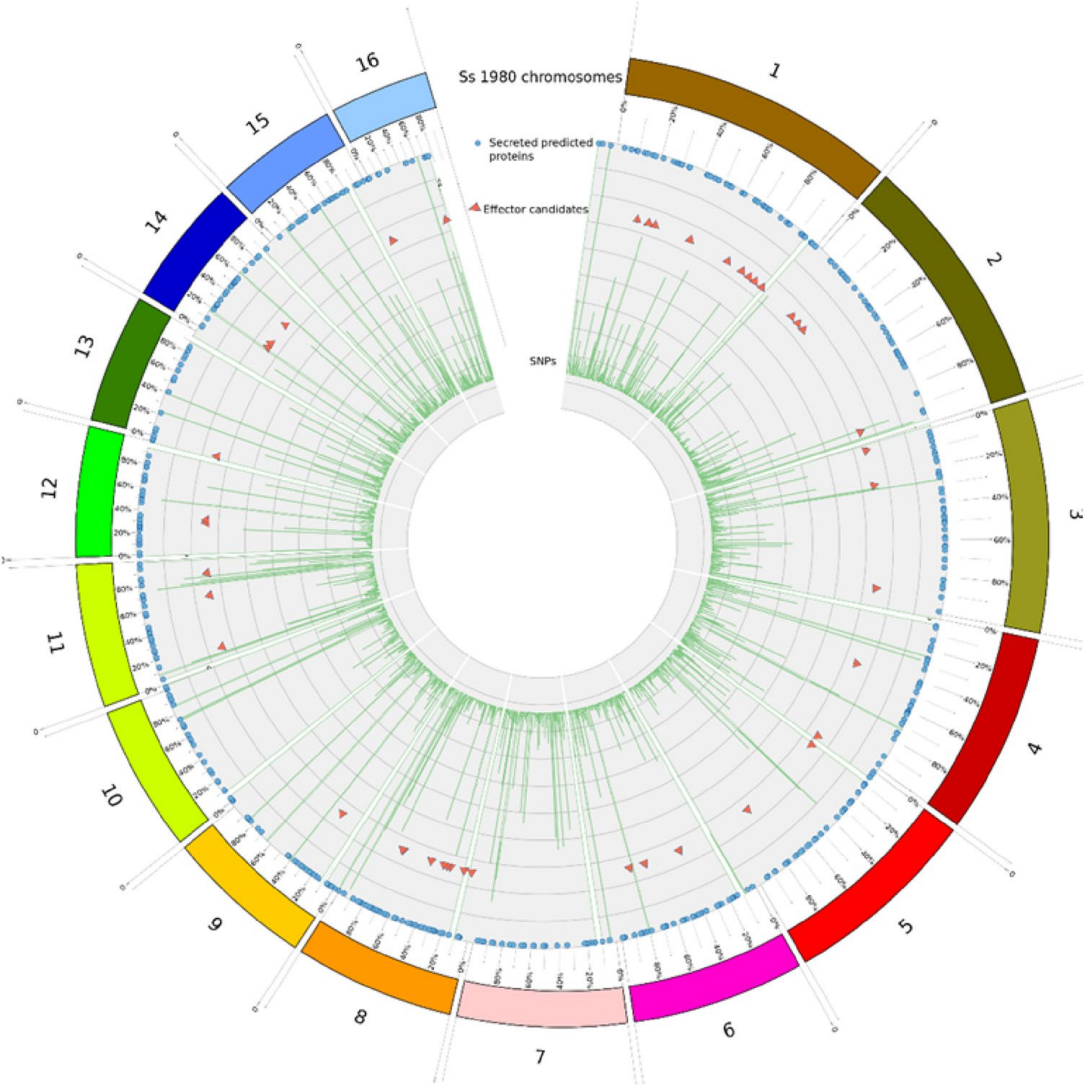
CIRCOS plot of the assembled scaffolds of ‘ESR-01’ against ‘1980’ isolate of *S. sclerotiorum* displaying comparative genomic and secretory proteins features. Schematic representation of the total SNPs, secretory proteins, and the effector proteins of the secretome data of the *S. sclerotiorum ‘*ESR-01’ genome shown as a histogram (different colors and types) and inner circle (blue) is total SNPs and the scattered circle is secretory proteins and scattered red triangle is the effector proteins predicted in ESR-01 isolate. The diagram was plotted using Circos.

The comparative graphical mapping of 1006 predicted secretory proteins, 57 effectors candidates with SNPs variations predicted in *S. sclerotiorum* ‘ESR-01’ against *S. sclerotiorum* ‘1980’ isolate was analyzed by using Circoletto, a visualization tool based on sequence similarity showed the intergenomic relationship between the two geographically distinct isolates of the same species (Fig. 6). The homology alignment using NCBI blast showed that 41 out of the predicted 57 effectors candidates in ‘ESR-01’, were completely aligned whereas 16 were not. The probable reason for the nonalignment of these putative 16 ECs is their belongingness to the 30 novel putative effector candidates whereas the remaining 14 of them were aligned as other the 27 annotated effectors. The chromosome-wise distribution of the predicted effectors showed the maximum numbers 9, 7, and 4 of them were present on Chromosomes 1, 8, and 2, respectively. Three effectors were found distributed on each of chromosomes 3, 6, 11, and 14 whereas two effectors were on each of chromosomes 9, 12, and 13. A single effector was found on each 9, 12, and 13 chromosomes whereas no effectors were present on chromosome numbers 7, 10, and 15 (Fig. 6). Overall, each of the predicted effectors of the ‘ESR-01’ isolate has multiple SNPs concerning the ‘1980’ isolate of the *S. sclerotiorum* as shown in Fig. 6. The even distribution pattern of the predicted secreted proteins of ‘ESR-01’ on 16 different chromosomes of *S. sclerotiorum* tells that not all secreted proteins will serve as effectors but all the effectors are the secretory proteins.

### Expression pattern of the effector candidate gene in *S. sclerotiorum*

**Figure 7:**
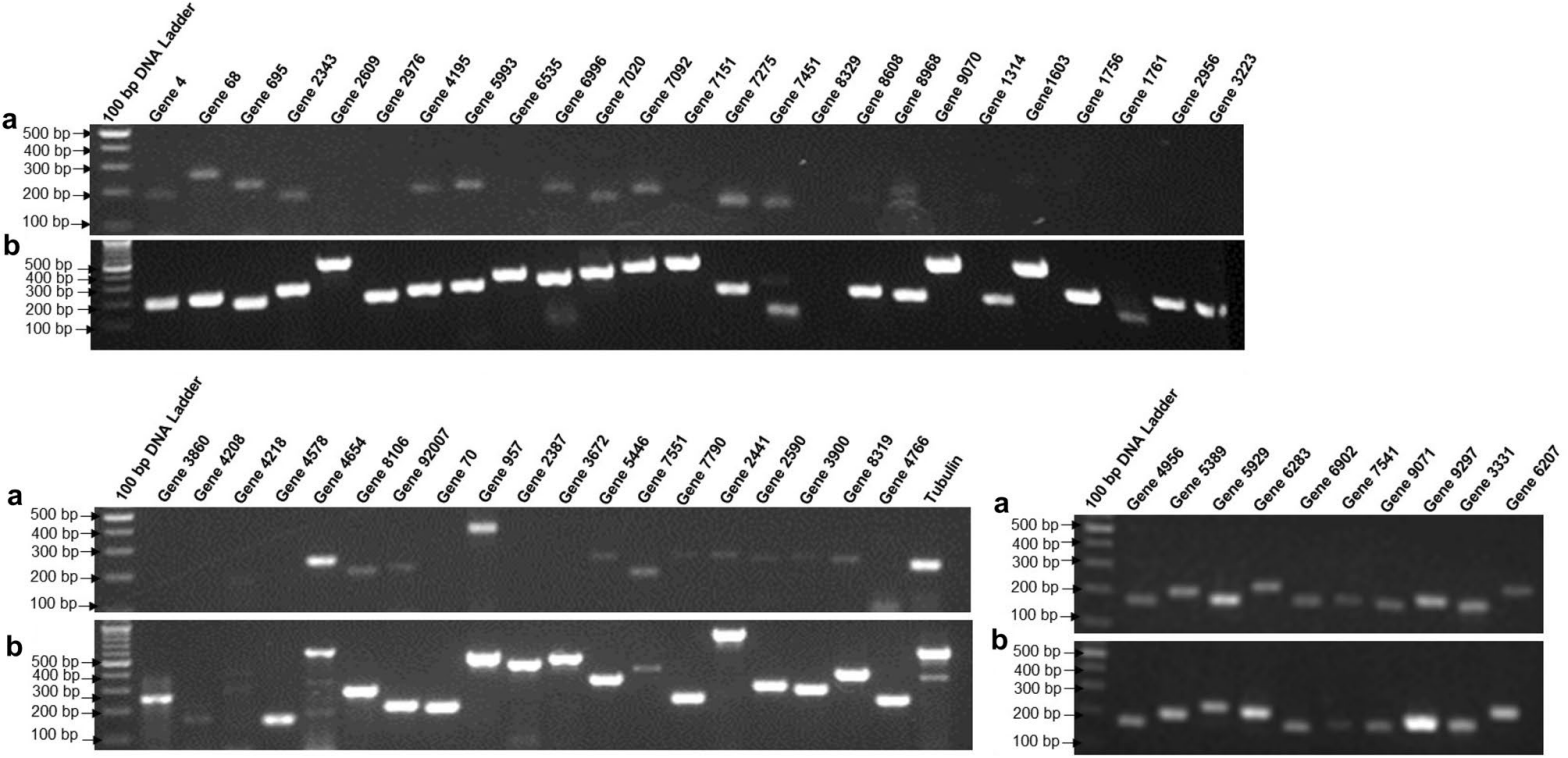
Expression profiling of the predicted effector molecules using (**a**) cDNA, and (**b**) DNA of *S. sclerotiorum ‘*ESR-01’ isolate as a template. Lanes: predicted effector candidates, M: 100 bp DNA ladder, and Tubulin, as control.

Expression analysis of predicted effector candidates was experimentally validated to ascertain their role in pathogenesis. In the expression analysis of all the 57 predicted *S. sclerotiorum* secretory effector proteins (SSEPs), 54 were shown they transcribed in cDNA of *S. sclerotiorum* ‘ESR-01’ isolate (Figs. 7, S2). Most of them have shown their functional relevance to pathogenesis like gene 70 (Isochorismatase hydrolase), gene 957 (Clock-controlled 6), gene 2387 (oxalate decarboxylase activity), gene 3672 (alpha beta-hydrolase), gene 5446 (Hydrolytic enzyme), gene 6207 (SCP-like extracellular), gene 7551 (Protease propeptide inhibitor), gene 7790 (Di-copper centre-containing), gene 2441 (2-deoxy-d-gluconate 3-dehydrogenase), gene 2590 (Phosphotyrosine phosphatase), gene 3900 (Short-chain dehydrogenase reductase family), gene 4766 (Proteasome subunit beta type-2), and gene 8319 (Related to iron-sulfur assembly 1). The presence of the double bands in some of the effectors like gene 8968 was probably due to the presence of different isoforms of the genes because of alternative splicing or the presence of different transcription start sites in the gene. (Fig. S3).

**Figure 8:**
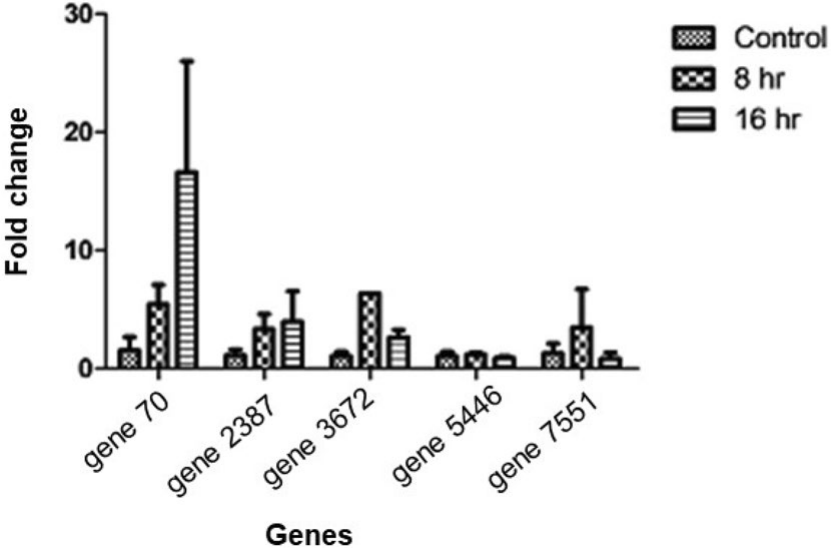
Expression analysis of the selected secretory effector genes by qRT-PCR in the *S. sclerotiorum* infected leaves of *B. juncea* after 8- and 16- hrs of infection.

Based on the functional relevance of the *S. sclerotiorum* secretory effector proteins (SSEPs) to pathogenesis, five of them were selected for qRT-PCR (Fig. 8) which showed the isochorismatase hydrolase (gene 70), and oxalate decarboxylase (gene 2387) was induced gradually in due course of infection whereas alpha, beta-hydrolase (gene 3672), and protease propeptide inhibitor (gene 7551) were induced at the early stage (up to 8 h) and downregulated at the later stage (16 h) of infection. One of the SSEPs with hydrolytic function (gene 5446) has shown neutral to the different stages of infection.

## Discussion

Economically destructive phytopathogen *S. sclerotiorum* causing stem rot disease in oilseed *Brassica* is a broad host range ascomycete fungus and it infects the host by overpowering the host defense system with their interacting virulence factors^16^. The life cycle of *S. sclerotiorum* has a very short biotrophic phase followed by a long necrotrophic phase which is also considered semi-biotrophs^17,18^. Hence, the pathogenesis mechanism of *S. sclerotiorum* is comparatively more complex than a simply necrotrophic or biotrophic fungus^11^. However, the recent advances in understanding the lifecycle and pathogenesis mechanism of some necrotrophic fungi suggest that the effector repertoire of the pathogen plays important role in establishing infection by suppressing the host defense, evading the hypersensitive response that concurrent with the initial events similar to the biotrophic mode of infection^16,19,20^. Effector repertoire protects the pathogen from the host’s oxidative bursts after infection^21^ and induces host cell death. Rapid adaptation to a broad host range could also be due to the presence of repeat elements, which may cause necessary genetic variation. Microsatellites (SSR) markers are a promising and rapid tool to decipher molecular recognition and in identifying the genetic differentiation among the isolates of the pathogen having differential virulence. Genome-wide variation analysis using SNPs could also reveal conserved and varied molecular machinery regulating the infectivity of the pathogen^22,23^.

The differential response of the host towards geographically distinct isolates made this necessary to dissect the various genetic elements and the genomic features of the pathogen. Whole-genome sequencing (~ 129 × coverage) of *S. sclerotiorum* “ESR-01” isolate and its de novo assembly will facilitate of developing a comprehensive understanding of their genomic evaluation concerning its universality and broad-host-range of infection. The total assembly size was 40.98 Mb, which was 2.68 Mb larger than the pre-sequenced *S. sclerotiorum* “1980” isolate (38.3 Mb)^13,14^. However, the protein-coding genes obtained in *S. sclerotiorum* ‘ESR-01’ isolate were relatively less in number (9469) compared to those found in *S. sclerotiorum* “1980” (14,522)^13^ and re-sequenced genome of *S. sclerotiorum* “1980” (11,130)^14^. The lower number of the genes in ‘ESR-01’ may be due to the difference in the origin of isolates or a result of the stringent gene prediction methodology adopted to minimize the redundant genes prediction.

Annotation results of the predicted genes showed a higher percentage of them are involved in cell and cellular components development, cellular processes, and metabolic processes that serve as the building blocks for the fast-growing pathogen. Further, the KEGG pathway analysis revealed a similar pattern of distribution where also a high number of genes were involved in protein-making machinery followed by carbohydrate metabolism and transport-related pathways required for basic life functions.

Repeat elements are the genomic feature that enables the pathogen to fast adapt to the varying hosts and changing environment. Mostly, repetitive DNA content spans 1–25% of a fungal genome^24^. The percentage of repeat elements within the genome and secretory effectors molecules determine the pathogen and effector evolution in the due course of time. DDE-1 is the most abundant transposable element found in *S. sclerotiorum* ESR-01 isolate. This family of repeat elements has a conserved motif with three amino acids D, D, and E making an active center of protein where D is aspartic acid and E is glutamic acid^25^.

The microsatellites studies in *S. sclerotiorum* ‘ESR-01’ isolate revealed that trinucleotide repeats are most abundant, occupying 66% of total SSRs identified. Among trinucleotide repeats, AAT/GAT/TCA was the most frequently (107/106/105-times) occurring element whereas GTT was the least frequent (27 times). The genome-wide studies showed a total of 27,450 SNPs were present, out of which T has been substituted around 14,081 times. SNPs showing transversion from T to C have occurred most frequently (4927 times) whereas T/G has occurred 961 times followed by transition T/A occurring 732 times.

The successful infection of the host to a fungal pathogen begins right from their interactions with the secretion of the proteinaceous effectors, hydrolytic enzymes, and various secondary metabolites during the process^26^. Secretory proteins play crucial roles at the early stage of the infection by facilitating pathogen colonization during host–pathogen interaction. Out of 554 predicted secretory proteins, 57 were filtered out as putative effector candidates based on the characteristic features of the fungal effector proteins. Secretome analysis of *S. sclerotiorum* ‘1980’ isolate by Guyon et al.^12^ predicted 745 secretory proteins resulting in 78 potential effector candidates whereas Derbyshire et al.^14^ predicted 523 secretory proteins, out of which 70 were predicted to be likely. The functional correlation of the predicted 369 secretory proteins in *S. sclerotiorum* ‘ESR-01’ isolate in pathogenesis was further evidenced by the presence of CAZymes in 164 proteins. The GO annotation of the secretory proteins suggests that the secretome of the ‘ESR-01’ isolate consists of diverse proteins other than CAZymes and most of the predicted effector candidates were involved in oxidoreductase activities and membrane-bound functions. The pathogenesis-related proteins of *S. sclerotiorum* “ESR-01” isolates in an effector repertoire comprised of hydrolases, oxidoreductases, peptidases, transferases, and isomerases. Most of these proteins attributed virulence to the pathogen. The specific functions of the predicted effector candidates in the ‘ESR-01’ isolate was explored with the literature described and validated their function. Three of the effector candidates (gene 70, gene 3672, gene 5446) among 27 annotated effectors viz*.* isochorismatase, alpha beta-hydrolase, and one another class of hydrolase revealed hydrolytic activity which is also evidenced to be important for virulence via differential expression in *S. sclerotiorum* transcriptome on *B. napus* infection^17,27^. One of the secretory effectors (gene 2387) is a cupin family protein showing oxalate decarboxylase activity. The functional evidence of oxalate decarboxylase in facilitating the early stage of infection by *S. sclerotiorum* through promoting the compound appressoria development has been laid by Liang et al.^8^. The gene 6207 and gene 7275 secretory effectors have shown SCP-like extracellular and peptidyl-Prolyl *cis*/*trans* isomerases (PPIases) activity. The former is a CAZyme having a CBM-5 domain (carbohydrate-binding modules) and categorized under the defense response to fungus whereas the latter was involved in protein folding and discovered in the PHI database against *B. cinerea* showing mixed response to pathogenicity. The small secreted protein, SCP-like extracellular peptide has been shown induced in rice on *Magnaporthe oryzae* infection^28^. The role of PPIases has also been established in virulence and as a potential drug target by Unal and Sterinert^29^ along with its known role in biological processes such as gene expression, protein secretion, and signal transduction. The role of PPIases in the virulence of *S. sclerotiorum* was evidenced due to its significant differential protein expression in Δ*SsNsd1* (a GATA-type IVb transcription factor) mutant-mediated abolishment of appressorium^30^. Other secreted proteins gene 7790, gene 3331, gene 3900, and gene 2441 showing oxidoreductase activity were di-copper center-containing proteins with esterase activity (RNA phosphodiester bond), Rieske domain-containing, short-chain dehydrogenase reductase family, and 2-deoxy-d-gluconate 3-dehydrogenase, respectively. Laccases are the di-copper or multi-copper compounds induced in *B. cinerea* during host tissue colonization^31^ and their role in melanin biosynthesis has also been reported while the characterization of cell wall proteomes^32^. The predicted CAZyme 2-deoxy-d-gluconate-3-dehydrogenase (gene 2441) which is enriched with glycosyltransferase 51 domain has earlier been categorized as lethal in PHI base against *Ralstonia solanacearum* whereas short-chain dehydrogenase reductase family protein (gene 3900) was categorized in reduced virulence as evidenced by experiments on *Penicillium expansum*. The 2-deoxy-d-gluconate 3-dehydrogenases are the pentose phosphate pathway enzyme that gets up-regulated in the presence of xylan in the growth media of *Neurospora crassa* depicting its association with hemicellulose degradation^33^. Predicted secretory effector phosphotyrosine phosphatase (gene 2590) was reported to be involved in the regulation of virulence and multi-stress tolerance in *B. cinerea*^34^ and found in PHI base as a reduced virulence category as experimented in *Burkholderia cenocepacia*. Small COPII coat GTPase SAR1 (gene 8968) is the predicted effector of *S. sclerotiorum* “ESR-01”. SAR GTPases (secretion-associated Ras-related protein) regulate vesicular transport by recruiting the coat protein complex II (COPII) assembly to generate transport vesicles^35^. In *Trichoderma reesei,* GTPase SAR1 recruits COP-II vesicle to transport secretory proteins through vesicular transport, and also proteomic analysis of these extracellular vesicles revealed the presence of CAZymes trigger through cellulose in growth media^36^. The other annotated secretory effectors include protease propeptide inhibitor (gene 7551), d-tyrosyl-tRNA deacetylase (gene 2609), Phospholipase A2 (gene 4192), phosphatidylglycerol phosphatidylinositol transfer (gene 5993), Sm-like ribonucleoprotein (gene 7451), proteasome subunit beta type-2 (gene 4766), RING-H2 zinc finger RHA1a-like (gene 8106), Mpv17 PMP22 family (gene 2343) and kinases (gene 6535). Among the 57 predicted effectors 30 were novel and unannotated those include necrosis and ethylene-inducing peptides (gene 7020, gene 7092), BYS1 and PAN domain-containing proteins (gene 7541, gene 9070), phospholipases (gene 6283), epimerases (gene 4208), dehydrogenases (gene 4195), and kinases (gene 1761) activities containing enzymes. Necrosis and ethylene-inducing peptides (gene 7020, gene 7092) and BYS1 domain-containing proteins (gene 7541) were also enriched with CBM-13 and AA11 domains of CAZymes. Among unannotated proteins, PHI prediction categorized probable short-chain dehydrogenases (gene 4195) and necrosis/ethylene inducing peptides (gene 7020, gene 7092) under unaffected pathogenicity searched against *Fusarium graminearum and Botrytis elliptica,* respectively*.* Three more unannotated protein genes 4, 7151, and 8329 were also predicted in the PHI database and were categorized into reduced virulence searched against *Magnaporthe oryzae.* Expression analysis of selected effector candidates showed their transcript profile in the *S. sclerotiorum* genome validated the predicted effector candidates. Out of these expressed SSEPs, gene 6207, and gene 2441 (SCP-like extracellular, 2-deoxy-d-gluconate-3-dehydrogenase) have CBM5 and GT51 classes of CAZymes respectively, present in them. Also, homology of gene 2590 (phosphotyrosine phosphatase) and gene 3900 (short-chain dehydrogenase reductase) was detected against *Burkholderia_cenocepacia* and *Penicillium_expansum* respectively to be categorized as reduced virulence in PHI-base while 2-deoxy-d-gluconate 3-dehydrogenase (gene 2441) was categorized as lethal detected against *Ralstonia solanacearum.*

Secondary metabolites are synthesized to perform various functions by pathogenic fungi such as virulence, defense, nutrient uptake, and signaling which tend to interfere with the host cell structure and functions^37^. The loss of the SM biosynthetic pathway has been found associated with the biotrophic life cycle in the fungal pathogen^37^. Many secondary metabolites like siderophores, pigments, and phytotoxins contribute to the virulence of pathogenic fungi by interfering with host cell structure and function^38^. Most often the genes encoding secondary metabolites are coregulated and clustered at one genomic locus called biosynthetic gene clusters (BGCs)^39^. These BGCs can be predicted based on homology with their key ‘backbone’ enzymes along with the ‘decorating’ enzymes for methylation, oxidation, reduction, or glycosylation using bioinformatics tools. Nonribosomal peptide synthases (NRPS) and polyketide synthases (PKS) form the key enzymes of BGCs. These clusters also include other genes like transcriptional regulators, precursor enzymes, and transporters^40^. In *B. cinerea* two of the PKS genes encoding botcinic acid were observed upregulated *in planta* of which one of them was found upregulated in *S. sclerotiorum* during the host infection in *B. napus*^17^. Graham-Taylor et al.^41^ have functionally characterized in vitro at 6-time points of infection by expression analysis of 11 secondary metabolites genes in *S. sclerotiorum* homologous to *B. cinerea*. Naphthalene is involved in the biosynthesis of 1,3,6,8-tetrahydroxynaphthalene (T4HN) which is converted to Dihydroxy naphthalene (DHN) melanin in sclerotia formation. Melanin is important for virulence as it builds the appropriate turgor pressure in appressoria for penetrating the host tissue along with the protection of cells from adverse environmental conditions^42,43^. The homologs of phytoene synthase were found upregulated in *S. sclerotiorum* and *B. cinerea* during infection of *B. napus*^17^.

The presence of the functionally diverse secretory effectors molecules, CAZymes, and PHI-related genes along with the other genomic features like SSRs, SNPs, and transposable elements function in an organized manner to establish pathogenesis over the host and makes the *S. sclerotiorum* pathosystem complex. We explore the various genomic features of the *S. sclerotiorum* pathogen and their involvement with the broad host range pathogenesis by using whole-genome sequence information and stringent bioinformatic tools to reveal genome-wide effector distribution and their functional relevance.

## Methods

### Strain, culture conditions, and genomic DNA isolation

ESR-01 is a high and consistently virulent isolate (Table 3) among the 65 geographical isolates of *S. sclerotiorum* infected *Brassica* plants from ten provinces in India^1^. The fungus, with off-white mycelial texture and brownish-black sclerotia forming in the peripheral pattern, was isolated from the infected *B. juncea* plants at Bharatpur (Long. 77.300° E; Lat. 27.150° N), Rajasthan, and was analyzed in this study. The 5 days old mycelial mat freshly grown on potato dextrose agar (PDA; HiMedia Laboratories, India) at 20 ± 2 °C was harvested and the DNA was extracted with 2% cetyl-trimethyl ammonium bromide (CTAB). 2 µL of extracted genomic DNA was resolved on 0.8% agarose gel and the quality and quantity were assessed by using the Nanodrop spectrophotometer (Thermo Scientific). Sample identity was verified with ITS sequence-specific primers (Table S1).

**Table 3:** The geographical origin, pathogenicity, growth, and morphological characteristics of the *S. sclerotiorum* ‘ESR-01’ isolate.

Strain	*Sclerotinia sclerotiorum* ‘ESR-01’		
Source of isolation	*B. juncea*		
Place of origin	Bharatpur, Rajasthan, India; 27° 15’ N; 77° 30’ E		
Virulence over the different *Brassica* species	*Brassica* species/cultivars	Lesion Length (cm)	Mean lesion size (cm)
	*B. juncea;* (NRCDR-2)	14.0	17.7
	*B. rapa, var* Toria; (Uttara)	18.7	
	*B. rapa, var yellow sarson;* (NRCYS 5-2)	21.0	
	*B. rapa’ var brown sarson*	17.0	
Mycelium	Growth on PDA after 72 h (mm) at 22 °C	85 (Petri plates of 20 × 90 mm size)	
	Growth on PDA after 96 h	90	
	Color	Whitish	
	Texture	Scattered	
Sclerotia	Initiation (Days)	04	
	Days of formation	05	
	Sclerotia/plate (90 mm)	40	
	Color	Black	
	Pattern	Peripheral (Spread)	
	Diameter (mm)	1.9	
	Length (mm)	3.6	

### Genome sequencing and assembly

**Figure 9:**
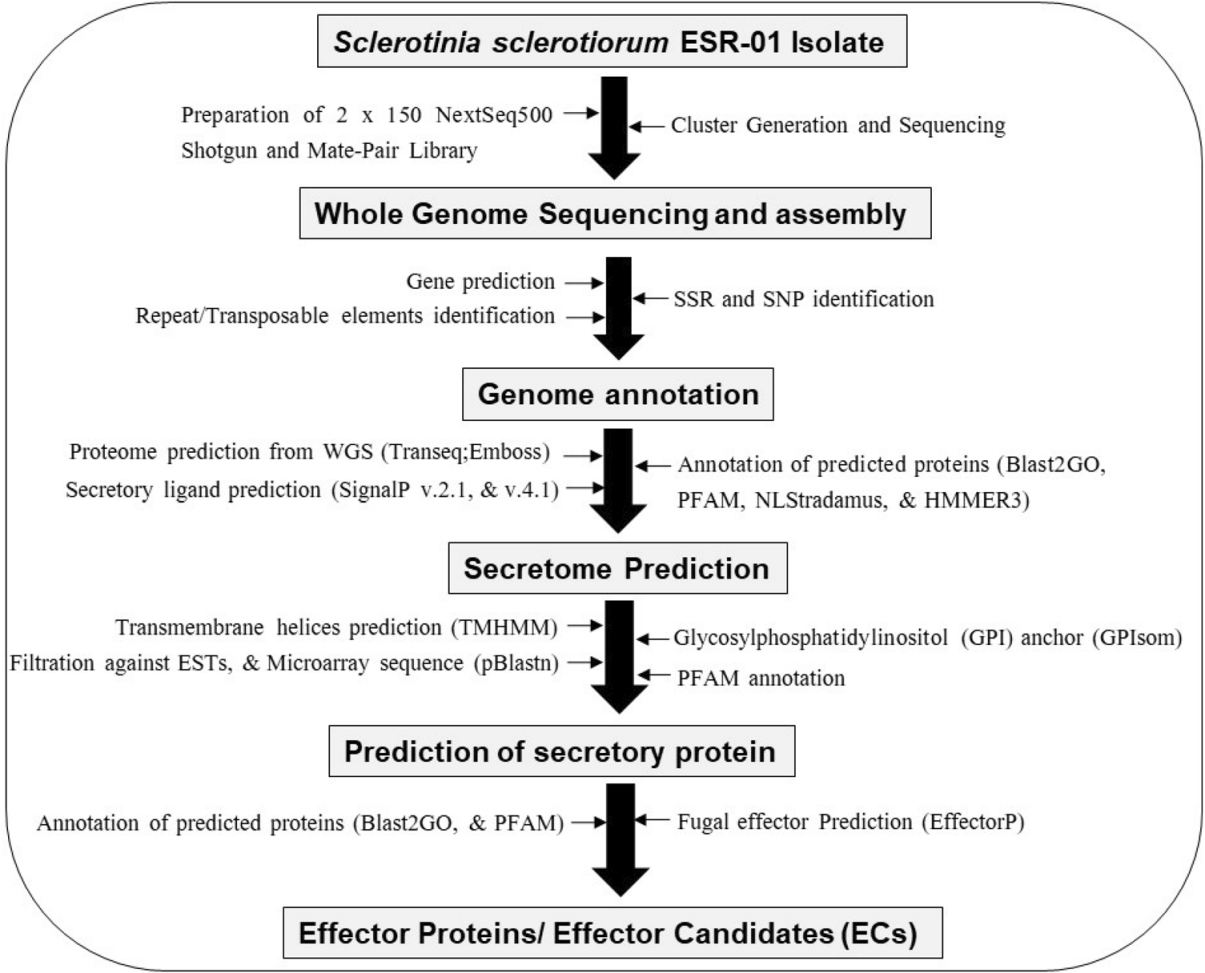
A schematic outline of the S. *sclerotiorum* ‘ESR-01’ whole genome sequencing and bioinformatic analysis.

The whole-genome sequencing and analysis of *S. sclerotiorum* ‘ESR-01’ isolate was done by using the Illumina NextSeq 500 platform following the pipeline as shown in Fig. 9. The shotgun paired-end library was prepared using the TruSeq Nano DNA library prep kit and the mate-pair sequencing library was prepared by the Illumina Nextera Mate Pair sample preparation kit. Quality assessment of the shotgun library and the mate-pair library using Agilent 4200 tape station showed a mean fragment size distribution of 478 bp and 818 bp, respectively. Both the libraries were sequenced on Illumina NextSeq 500 using 2 × 150 bp chemistry. The sequenced raw reads were trimmed using Trimmomatic v0.35 and low-quality bases having a quality threshold < 20 were removed from both ends. After trimming, the reads with lengths < 70 bp were discarded. The draft genome was assembled with the help of SOAPdenovo (v.2.04) using filtered high-quality paired-end (PE) and mate-pair (MP) reads into scaffolds with optimized *k*-mer 97.

### Gene prediction

Protein coding gene prediction was carried out with assembled scaffolds of *S. sclerotiorum* using the AUGUSTUS-3.2.1 program. *Botrytis cinerea* was used as a gene model to predict genes from the assembled scaffolds.

### Genome annotation

Functional annotation of predicted genes was performed by homology search against NCBI non-redundant (nr) protein database using BLASTX program (NCBI-blast-2.3.0 + standalone tool) with cut-off E-values of ≤ 1e−05 and identity ≥ 40%. Gene ontology (GO) analysis was performed using Blast2GO PRO. For pathway analysis, the protein sequences of the predicted genes were annotated from the Kyoto Encyclopedia of Genes and Genomes (KEGG)^44^ (Minoru and Goto 2000) using blastKOALA. Annotated protein sequences were assigned KEGG Orthology (KO) identifiers, corresponding Enzyme Commission (EC) numbers, and metabolic pathways of predicted CDS using KEGG automatic annotation server [KASS (http://www.genome.jp/kaas_main)]. Secondary metabolite clusters prediction was based on searching genes encoding backbone enzymes and other protein domains associated with clusters using the fungal version of AntiSMASH v.6.0^45^.

### Transposable elements, SSR, and SNP identification

Repeat elements from assembled scaffold were identified by using the TransposonPSI analysis tool (http://transposonpsi.sourceforge.net/). It finds the homology of scaffold to proteins encoded by diverse families of transposable elements. PSI-Blast was used with a collection of (retro-) transposon ORF homology profiles to identify statistically significant alignments in scaffold sequence.

The high-throughput simple sequence repeat (SSR) search to identify di- to hexanucleotide SSR motifs was performed using the MicroSAtellite Identification tool (MISA v 1.0; http://pgrc.ipk-gatersleben.de/misa/download/misa.pl) from assembled scaffolds with default parameters. The potential SSRs were identified as ranging from dinucleotide motifs with a minimum of ten repeats to trinucleotide motifs with 3 repeats while tetra, penta, and hexanucleotide motifs with a minimum of five repeats along with interruption of 200 bp allowed between two SSRs.

The single nucleotide polymorphisms (SNPs) were predicted by aligning the high-quality reads of ESR-01 with the available *Sclerotinia sclerotiorum* '1980' strain (ATCC18683) genome sequence using a BWA-mem aligner. The resulting sequence alignment/map (SAM) file was converted into stored binary alignment/map (BAM) files using the software Samtools (v 0.1.18). The mpileup program which is incorporated in Samtools was used to make a .vcf file (variant call format) from the bam file. The SNPs were filtered based on the read depth of 15 and flanking of 150 bp.

### CAZymes prediction and potential pathogenicity-related protein identification

CAZymes prediction and classification were performed using CAZyDB (carbohydrate-active enZymes database). Potential pathogenicity-related proteins were identified from predicted genes by homology search against the Pathogen-Host Interaction database (PHI-base) using BlastX search with a threshold e-value of ≤ 1e−05. PHI-base catalogs experimentally verified pathogenicity, virulence, and effector genes from fungal, oomycete, and bacterial pathogens.

### Secretome prediction

The predicted gene sequences were translated into protein sequences by using Transeq (Emboss) standalone version and searched against the secretory proteins of *S. sclerotiorum* from Fungal Secretome KnowledgeBase (FunSecKB, http://bioinformatics.ysu.edu/secretomes/fungi.php) using BlastX at e-value threshold 1e−05. The candidate effector proteins were predicted by using the following channel: i) Signal IP 2.1^46^ and Signal IP 4.1^47^ was used to identify the proteins containing secretory signals. The method incorporates a prediction of cleavage sites and a signal peptide/non-signal peptide prediction based on a combination of several artificial neural networks. (ii) TMHMM v.2.0^48^ was used to predict transmembrane helices. In this the protein-containing one transmembrane domain after the first 60 amino acids or at least two transmembrane domains in total, was labeled as a putative membrane-bound protein and were filtered out from those containing secretory signals (iii) Remaining proteins were subjected to GPIsom (https://www.gpi.unibe.ch/)^49^ for predicting GPI (glycophosphatidylinositol) membrane-anchoring sequence to filter out along with the unclassified proteins (iv) pBLASTN search was used to identify the remaining secretory proteins for having similarity to known fungal proteins by filtering against EST and microarray sequences of *B. napus* after *S. sclerotiorum* infection, (v) Predicted secretory proteins were subjected to pFAM and Blastp annotation^50^ with e-value threshold ≤ 0.05)^51^, (vi) EffectorP v.1.0 (http://effectorp.csiro.au/)^52^ was used for predicting the effector candidates to distinguish the secretory proteins from secreted effectors candidates in *S. sclerotiorum* pathogen.

### Secretome annotation and analysis

Secreted effectors were functionally annotated by assigning GO terms using BLAS2GO. They were classified into two categories one having a complete description and the other ones are the novel ones that have not yet been annotated. For pathway analysis, the protein sequences of the predicted genes were annotated from the Kyoto Encyclopedia of Genes and Genomes (KEGG) as above. BLASTP analysis of all secretory proteins, as well as secreted effectors, was carried out against the Pathogen-Host Interaction database (PHI-base) with a threshold e-value of ≤ 1e−05 to categorize them as hypervirulence, reduced virulence, loss of pathogenicity, and others. Carbohydrate-active enzymes (CAZymes) were analyzed in all the secretory proteins and secreted effectors using CAZyDB (carbohydrate-active enZymes database). The graphical mapping of all secretory proteins secreted effectors, and SNPs was performed using the CIRCOS visualization tool version 0.69 for presenting relationships among them.

### Transcript analysis of the predicted effector genes in *S. sclerotiorum*

The mycelium of *S. sclerotiorum* grown for a week in 250 ml Potato Dextrose Broth (PDB) medium with continuous shaking at 80 rpm and 20 ± 2 °C was filtered using an autoclaved muslin cloth and used for RNA extraction by using the trizol Reagent (Invitrogen). For this mycelium was homogenized with liquid nitrogen in a pre-chilled mortar and pestle and 2 ml of trizol was added to the 100 mg powdered samples. Once the slurry thawed, it was transferred to two 2 ml centrifuge tubes and after 5 min at room temperature (RT), 300 μl of chloroform per ml of trizol reagent was added and mixed well by vigorously shaking for 15 s and was allowed to stand at RT for 2–3 min. The sample was then centrifuged at 12,000 × g for 15 min at 4 °C. The sample was subjected to a second chloroform extraction as above. The supernatant was transferred to a clean 1.5 ml centrifuge tube and 500 μl of chilled isopropanol was added and kept at − 20 °C for 1 h to precipitate RNA. Precipitated RNA was collected by centrifugation at 12,000 × g for 15 min at 4 °C. The pellet was washed with 1 ml of ice-cold 70% ethanol and vortexed briefly. Centrifugation at 7500 × g was carried out for 5 min to pellet RNA and then air-dried under Laminar Air Flow for 10–15 min. The RNA pellets were dissolved in 40 μl of nuclease-free water and mixed by gently tapping. RNA concentration and purity were measured using a NanoDrop Spectrophotometer (Nanodrop2000, Thermo Scientific). The integrity of the isolated RNA was assessed on agarose gel [1.2% agarose, 6% formaldehyde in 1 × MOPS buffer (20 mM 3-[N-morpholino] propane-sulfonic acid, 5 mM sodium acetate, 1 mM EDTA)]. DNA contamination in the isolated RNA was removed by DNaseI (Promega) treatment as per the manufacturer protocol.

For reverse transcriptase-polymerase chain reaction (RT-PCR), 2 μg of treated RNA was subjected to cDNA preparation using Applied Biosystems, a High-capacity Reverse transcription kit as per the manufacturer’s protocol. cDNA was used as a template for PCR using the primer sets for the randomly selected effector candidate genes, designed using the IDT oligo analyzer^2^ at default settings.

#### Quantitative expression of the predicted effectors

The mature leaves harvested from the 65 days old *Brassica* plants were inoculated with young mycelial plug of *S. sclerotiorum* ‘ESR-01’ isolate under the controlled condition as described by Gupta et al.^53^. The RNA was isolated from the control and inoculated leaf samples after 8- and 16 h of infection. The cDNA was prepared as described in the previous section. Real-time qRT-PCR was conducted using PowerUp™ SYBR™ Green Master Mix (Thermo Fisher Scientific Applied Biosystems), with Applied Biosystems StepOne Plus Real-Time detection system (Invitrogen, USA) using gene-specific (Table 4). The relative gene expression was calculated using the 2^−ΔΔCt^ where tubulin was taken as an internal transcript control. Each gene expression level was quantified using three biological and two technical replicates each. Two-step qRT-PCR reaction conditions were as follows: 95 °C for 10 min, followed by 40 cycles of 95 °C for 15 s; 60 °C for 1 min. This was followed by a Melt curve step consisting of 95 °C for 15 s, followed by 60 °C for 1 min; 95 °C for 15 s.

**Table 4:** List of primers used for quantitative expression analysis of the selected *S. sclerotiorum* effector proteins.

S. No	Oligos	Sequences (5ʹ- 3ʹ)	Tm (°C)	Amplicon length (bp)
1	Gene 70	F: CGTCATCGCCGTTGAAGGATTCAC R: GATGGCTGTTTGGACCTTGTTCAGC	62	215
2	Gene 2387	F: CGCATGGGACGAATTTGTGGTGG R: CCAGGCACGCTGCAGATCCAATTA	62	398
3	Gene 3672	F: AACCGTCTCGGCGATCATAACC R: ACCCGCAGTAGGAACTTCTTGTTG	62	249
4	Gene 5446	F: TGGACAGGAACGGTTGGAAGT R: CTACAACCATCGATTCCGCCAAG	62	248
5	Gene 7551	F: CAGCTGCTTTAGGCACAGTGTTG R: CGACTGGACAGTTGCAAGGACCTG	62	223

## Conclusion

In summary, the present study has opened new prospects for the comprehensive genomic study of a variety of biological processes that make *S. sclerotiorum* a successful necrotrophic polyphagous pathogen. Detailed comparative genomics studies may provide unexpected new insight into biological phenomena of general interest. Functional characterization of potential effector candidates is a prerequisite for determining their roles in pathogenesis. Such studies will provide insight and help in designing strategies to control the menace of *Sclerotinia* stem rot and other devastating fungal diseases in the crop of agricultural importance.

## Supplementary Information

Supplementary Information.

## Data Availability

The datasets generated and/or analyzed during the current study are available in the DDBJ/ENA/GenBank repository, under the accession JAGTAE000000000″. The version described in this paper is version JAGTAE010000000.
