# Supplementary material for: Draft genome sequencing and secretome profiling of Sclerotinia sclerotiorum revealed effector repertoire diversity and allied broad-host range necrotrophy
Source: Sci Rep. 2022 Dec 17;12:21855. doi: 10.1038/s41598-022-22028-z (PMC9759525; doi:10.1038/s41598-022-22028-z)
Supplement: Supplementary file 1 — Supplementary Information. [file 41598_2022_22028_MOESM1_ESM.pdf]

Table S1. Details of the primers used for identification of Sclerotinia species.

Primer	Primer Sequence (5′-3′)	Amplification size	Reference
ITS4	TCCTCCGCTTATTGATATGC	600 bp	White et al., 1990
ITS5	GGAAGTAAAAGTCGTAACAAGG		

Table S2. Assembly Statistics

Assembly Statistics	
No of Scaffolds	328
Total no of bases (bp)	40,981,405
Average Scaffold Size (bp)	124,943
Scaffold N50 (bp)	447,128
Max Scaffold Size (bp)	2,434,613
Min. Scaffold Size (bp)	500
Scaffold mean Size (bp)	124943.3
GC content %	37.71

Table S3. Scaffold Length Distribution

SSF-Brassica Sample Scaffold Size Range	No of Scaffold
1000≤Scaffold <5000	64
5000≤Scaffold <10000	21
10000≤Scaffold <20000	17
20000≤Scaffold <30000	7
30000≤Scaffold <40000	6
40000≤Scaffold <50000	11
Scaffold ≥50000	116

Table S4. Gene Length Distribution

SSF-Brassica Sample Scaffold Size Range	No of Genes
100≤Scaffold <500	861
500≤ Scaffold<1000	2404
1000≤Scaffold <5000	6019
5000≤Scaffold <10000	175
Scaffold≥10000	185

Table S5: List of the predicted secondary metabolite gene clusters in *S. sclerotiorum* 'ESR-01' isolate with the number of their constituent genes, coordinates in the genome, and their description.

Scaffold	Cluster	Type	From	To	Most similar	Genes in the

					known cluster	cluster
Scaffold 3	1	T1PKS	98632	146488	Botcinic acid	16
Scaffold 5	1	NRPS	639940	690441		
Scaffold 11	1	T1PKS	26772	73312	Naphthalene	9
Scaffold 21	1	NRPS	685260	729888		
Scaffold 29	1	NRPS	286836	330644		
Scaffold 30	1	NRPS-like	38012	81894		
Scaffold 47	1	Terpene	621321	642922	Squalestatin S1	5
Scaffold 60	1	NRPS	611075	655361		
Scaffold 62	1	NRPS-like	162733	205434		
Scaffold 77	1	T1PKS	7551	52220		
Scaffold 78	1	NRPS-like	386621	430352		
Scaffold 107	1	NRPS-like	79583	122006		
Scaffold 121	1	T1PKS	115423	162885		
Scaffold 139	1	NRPS-like	22029	65389		
Scaffold 171	1	T1PKS	1	39888		

Table S6: List of the predicted effector candidates in the *S. sclerotiorum* 'ESR-01' isolate genome.

S.N.	Genes	Enzyme names	Length (aa)	Description	InterPro IDs
S. sclerotiorum **effector candidates with their annotation**					
1	Gene 70	Isochorismatase hydrolase	219	P: Metabolic process; F: hydrolase activity	IPR000868 (PFAM)
2	Gene 957	Clock-controlled 6	212	P: Fungal-type cell wall organization; C: Membrane; F: Receptor activity; P: transport	IPR036942
3	Gene 1603	Pc22g06530 [*Penicillium rubens* Wisconsin 54-1255]	83	C: Membrane	No IPS match
4	Gene 2387	Cupin family	176	P: Oxalate metabolic process; F: Oxalate decarboxylase activity; F: metal ion binding; C: Cell wall;	IPR006045 (PFAM)
5	Gene 2609	D-tyrosyl-tRNA deacylase	145	F: D-tyrosyl-tRNA (Tyr) deacylase activity; F: Peptidyl-prolyl cis-trans isomerase activity; C: Cytoplasm; P: regulation of translational fidelity	IPR003732 (PFAM)

6	Gene 2976	Predicted protein	89	C: Extracellular region	IPR010636 (PFAM)
7	Gene 3672	Alpha beta-Hydrolase	167	F: Hydrolase activity	No IPS match
8	Gene 4192	Phospholipase A2	265	F: Phospholipase A2 activity; Calcium ion binding; P: Lipid catabolic process; C: Membrane; Extracellular	No IPS match
9	Gene 5446	Hydrolytic enzyme	125	P: Biological process; C: Cellular component; F: Molecular function	SignalP-TM (SIGNALP_GRAM_POSITIVE)
10	Gene 5993	Phosphatidylglycerol phosphatidylinositol transfer	153	C: Fungal-type vacuole lumen; P: Intracellular sterol transport	IPR003172 (PFAM)
11	Gene 6207	SCP-like extracellular	113	P: Defence response to fungus; defence response to bacterium	IPR009009 (PFAM)
12	Gene 6535	Kinase	178	F: ATP binding; Protein kinase activity; P: protein phosphorylation	IPR000719 (PFAM)
13	Gene 6996	RNA recognition motif containing	183	F: Nucleotide-binding; nucleic acid binding	IPR000504 (PFAM)
14	Gene 7275	Peptidylprolyl isomerase	183	C: Endoplasmic reticulum membrane; F: FK506 binding; P: Chaperone-mediated protein folding; protein peptidyl-prolyl isomerization	IPR001179 (PFAM)
15	Gene 7551	Protease propeptide inhibitor	78	F: Endopeptidase inhibitor activity; P: Proteolysis; negative regulation of endopeptidase activity C: Integral component of membrane; fungal-type vacuole	IPR037045 (G3DSA:3.30.70. GENE3D)

16	Gene 7790	Di-copper centre-containing	107	F: RNA binding; iron ion binding; oxidoreductase activity; ribonuclease T2 activity, P: oxidation-reduction process; RNA phosphodiester bond hydrolysis, endonucleolytic	No IPS match
17	Gene 8106	RING-H2 zinc finger RHA1a-like	168	F: Zinc ion binding; metal ion binding	IPR013083 (G3DSA:3.30.40. GENE3D)
18	Gene 8968	Small COPII coat GTPase SAR1	177	F: GTP binding; GTPase activity, P: ER to Golgi vesicle-mediated transport; Nuclear envelope organization; regulation of COPII vesicle coating, C: Endoplasmic reticulum membrane; exit site; Golgi membrane; COPII vesicle coat.	IPR006689 (PFAM)
19	Gene 2343	Mpv17 PMP22 family	187	P: Ethanol metabolic process, C: Mitochondrial inner membrane; integral component of membrane.	IPR007248 (PFAM)
20	Gene 2441	2-deoxy-d-gluconate 3-dehydrogenase	278	F: Oxidoreductase activity; P: Oxidation-reduction process	IPR002347 (PFAM)
21	Gene 2590	Phosphotyrosine phosphatase	164	F: Non-membrane spanning protein tyrosine phosphatase activity; C: cytoplasm; P: peptidyl-tyrosine dephosphorylation	IPR023485 (PFAM)
22	Gene 3331	Rieske domain-containing	109	F: Oxidoreductase activity; metal ion binding; 2 iron, 2 sulfur cluster binding, P: oxidation-reduction process.	No IPS match
23	Gene 3900	Short-chain dehydrogenase	256	F: Oxidoreductase activity; P: Oxidation-reduction process	IPR002347 (PFAM)

		reductase family			
24	Gene 4766	Proteasome subunit beta type-2	261	F: endopeptidase activator activity; P: proteasome-mediated ubiquitin-dependent protein catabolic process; positive regulation of endopeptidase activity, C: Endoplasmic reticulum membrane; proteasome core complex, beta-subunit complex.	IPR001353 (PFAM)
25	Gene 7451	Sm-like ribonucleoprotein	122	F: U6 snRNA binding; C: U4/U6 x U5 tri-snRNP complex; P: nuclear-transcribed mRNA catabolic process; P: cytoplasmic mRNA processing body assembly; spliceosomal snRNP assembly	IPR001163 (PFAM)
26	Gene 8319	Related to iron sulphur assembly 1	245	F: ferrous iron binding; structural molecule activity; 4 iron, 4 sulfur cluster binding; 2 iron, 2 sulfur cluster binding, P: biotin biosynthetic process; protein maturation by iron-sulfur cluster transfer, C: Mitochondrial matrix,	IPR000361 (PFAM)
27	Gene 8608	Hypothetical protein SS1G_10813	188	P: Cristae formation; C: mitochondrial crista junction; MICOS complex	IPR031463 (PFAM)
***S. sclerotiorum* effector candidates without annotation.**					
28	Gene 4	Cell surface	223		IPR021476 (PFAM)
29	Gene 68	Hypothetical protein sscle_08g062090	189		No IPS match
30	Gene 127	[*Botrytis cinerea*]	154		No IPS match
31	Gene 695	Cupin family	162		IPR009327

					(PFAM)
32	Gene 1314	Predicted protein	60		Mobidb-lite
33	Gene 1756	Predicted protein	195		Mobidb-lite
34	Gene 1761	Cyclin-dependent serine threonine-kinase, DDB_G0272797 DDB_G0274007-	163		No IPS match
35	Gene 2956	[*Botrytis cinerea*]	355		No IPS match
36	Gene 3223	Predicted protein	180		No IPS match
37	Gene 3860	Hypothetical protein sscle_09g074030	164		PTHR40845
38	Gene 4208	Nucleoside-diphosphate-sugar epimerase	218		No IPS match
39	Gene 4218	Predicted protein	185		No IPS match
40	Gene 4578	[*Botrytis cinerea*]	155		No IPS match
41	Gene 4654	[*Botrytis cinerea*]	134		No IPS match
42	Gene 4956	Hypothetical protein SS1G_05938	194		No IPS match
43	Gene 5389	[*Botrytis cinerea*]	192		No IPS match
44	Gene 5929	Hypothetical protein SS1G_13910	51		Mobidb-lite
45	Gene 6283	Phospholipase a2	279		Mobidb-lite
46	Gene 6902	Hypothetical protein sscle_01g004010	130		No IPS match
47	Gene 7020	Necrosis and ethylene inducing peptide 1	230		IPR008701 (PFAM)

48	Gene 7092	Necrosis and ethylene inducing peptide 1	226		IPR008701 (PFAM)
49	Gene 7151	Related to gEgh 16	283		IPR021476 (PFAM)
50	Gene 7541	BYS1 domain-containing	187		IPR037176 (G3DSA)
51	Gene 9070	PAN domain containing	343		IPR003609 (PFAM)
52	Gene 9071	Hypothetical protein SS1G_01426	218		No IPS match
53	Gene 9105	[*Botrytis cinerea*]	150		No IPS match
54	Gene 9200	Hypothetical protein sscle_11g081720	201		No IPS match
55	Gene 9297	Hypothetical protein sscle_02g022120	169		No IPS match
56	Gene 4195	Probable short chain dehydrogenase	248		IPR002347 (PFAM)
57	Gene 8329	-like family domain-containing 1	236		IPR008030 (PFAM)

Table S7. Functional annotations of the *S. sclerotiorum* isolate 'ESR-01' effector candidates and summary of the CAZymes categories.

**S. N.**	**CAZyme categories**	***S. sclerotiorum*** **'ESR-01' isolate genome**	***S. sclerotiorum*** **'ESR-01' isolate SPEPs**	***S. sclerotiorum*** **'ESR-01' isolate Effector Candidates**

1.	Glycosyl Transferases	542	12	1
2.	Glycosyl Hydrolases	550	77	-
3.	Auxiliary Activities	113	22	1
4.	Carbohydrate-Binding Module	167	22	3
5.	Polysaccharide Lyases	15	2	-
6.	Carbohydrate Esterases	119	22	-

Table S8. The potential pathogenicity-related genes of *S. sclerotiorum* isolate 'ESR-01'.

**Description**	***S. sclerotiorum* 'ESR-01' isolate genome**	***S. sclerotiorum* 'ESR-01' isolate SPEPs**	***S. sclerotiorum* 'ESR-01' isolate effector Candidates**
RV: Reduced Virulence	1094	67	5
LP: Loss of Pathogenicity	234	5	0
MO: Mix Outcome	207	17	1
L: Lethal	114	2	1
IV: Increased Virulence	42	2	0
O: Others	1074	60	4
Total	2765	153	11

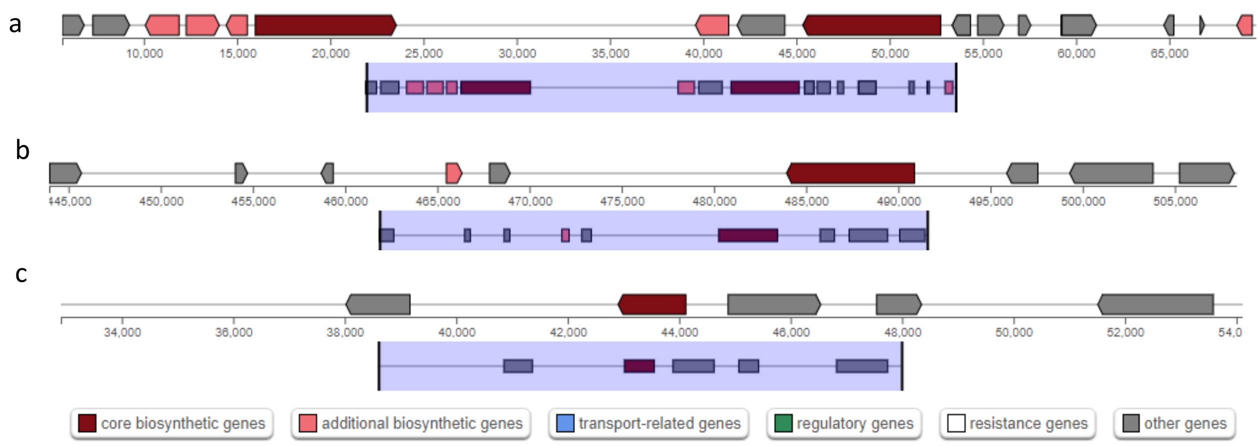

Figure S1: The diagrammatic representation of the gene clusters identified through AntiSmash analysis in the genomic scaffolds of the *S. sclerotiorum* 'ESR-01' isolate. (a) Botcinic acid, 16 gene cluster, (b) Naphthalene, 9 gene cluster, and (c) Squalestatin S, 5 gene cluster.

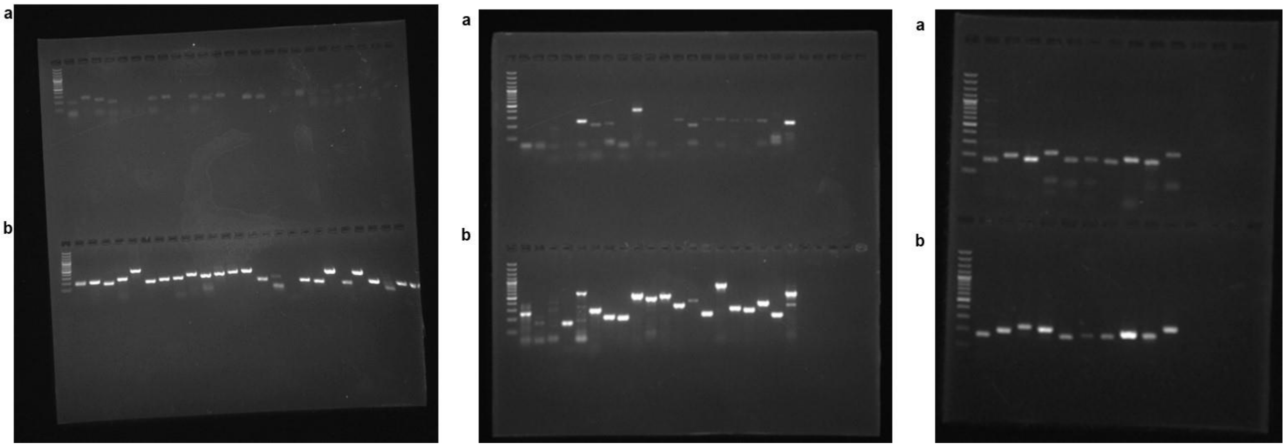

Figure S2: Expression profiling of the predicted effector molecules using (a) cDNA, and (b) DNA of *S. sclerotiorum* 'ESR-01' isolate as a template. Lanes: predicted effector candidates, M: 100 bp DNA ladder, and Tubulin, as control.

```
>Gene2590_Scaffold_24
FGENESH 2.6 Prediction of potential genes in
Sclerotinia_sclerotiorum genomic DNA
 Time:   Thu Nov 18 12:25:44 2021
 Seq name: Gene2590_Scaffold_24
 Length of sequence: 495
 Number of predicted genes 1: in +chain 1, in -chain 0.
 Number of predicted exons 1: in +chain 1, in -chain 0.
 Positions of predicted genes and exons: Variant   1 from   1,
Score:30.549585
    G Str   Feature   Start        End    Score          ORF           Len

   1 +    1 CDSo         1 -       495   38.52         1 -       495    495

********************* NetGene2 v. 2.4 ***********************

The sequence: Gene2590_Scaffold_24 has the following composition:

Length: 495 nucleotides.
26.1% A, 18.2% C, 25.1% G, 30.7% T, 0.0% X, 43.2% G+C

Donor splice sites, direct strand
---------------------------------
            pos 5'->3'  phase strand  confidence  5'      exon intron
3'
                235       0     +        0.87
GGCTCGAAAG^GTTTCTATCA

Donor splice sites, complement strand
-------------------------------------
   No donor site predictions above threshold.

Acceptor splice sites, direct strand
------------------------------------
            pos 5'->3'  phase strand  confidence  5'    intron exon
3'
                313       1     +        0.61
TTATCCTTAG^GGAGGAGAAC

Acceptor splice sites, complement strand
----------------------------------------
   pos 3'->5'  pos 5'->3'  phase strand  confidence  5'    intron exon
3'
      213         283       1     -        0.54
ATGCTTATAG^TCACTAATCC

--------------------------------------------------------------------------
---

CUTOFF values used for confidence:

Highly confident donor sites (H):     95.0 %
Nearly all true donor sites:          50.0 %

Highly confident acceptor sites (H):  95.0 %
Nearly all true acceptor sites:       20.0 %
```

Figure S3: Prediction of the exons, introns and the possible alternatively spliced form of the genes. Gene 2590 has the single intron of the 78 nucleotide (position 285-313).
